# When Nothing Turns Itself Inside out and Becomes Something: Coating Poly(Lactic-*Co*-Glycolic Acid) Spheres with Hydroxyapatite Nanoparticles vs. the Other Way Around

**DOI:** 10.3390/jfb13030102

**Published:** 2022-07-23

**Authors:** Vuk Uskoković, Victoria M. Wu

**Affiliations:** 1 TardigradeNano LLC., 7 Park Vista, Irvine, CA 92604, USA; victoria.wu@tardigradenano.com; 2 Department of Mechanical Engineering, San Diego State University, 5500 Campanile Dr., San Diego, CA 92182, USA

**Keywords:** bone, calcium phosphate, core/shell particles, drug delivery, nanocomposite, zeta potential

## Abstract

To stabilize drugs physisorbed on the surface of hydroxyapatite (HAp) nanoparticles and prevent burst release, these nanoparticles are commonly coated with polymers. Bioactive HAp, however, becomes shielded from the surface of such core/shell entities, which partially defeats the purpose of using it. The goal of this study was to assess the biological and pharmacokinetic effects of inverting this classical core/shell structure by coating poly(lactic-*co*-glycolic acid) (PLGA) spheres with HAp nanoparticles. The HAp shell did not hinder the release of vancomycin; rather, it increased the release rate to a minor degree, compared to that from undecorated PLGA spheres. The decoration of PLGA spheres with HAp induced lesser mineral deposition and lesser upregulation of osteogenic markers compared to those induced by the composite particles where HAp nanoparticles were embedded inside the PLGA spheres. This was explained by homeostatic mechanisms governing the cell metabolism, which ensure than the sensation of a product of this metabolism in the cell interior or exterior is met with the reduction in the metabolic activity. The antagonistic relationship between proliferation and bone production was demonstrated by the higher proliferation rate of cells challenged with HAp-coated PLGA spheres than of those treated with PLGA-coated HAp. It is concluded that the overwhelmingly positive response of tissues to HAp-coated biomaterials for bone replacement is unlikely to be due to the direct induction of new bone growth in osteoblasts adhering to the HAp coating. Rather, these positive effects are consequential to more elementary aspects of cell attachment, mechanotransduction, and growth at the site of contact between the HAp-coated material and the tissue.

## 1. Introduction

Nanocomposites represent a most prospective category of materials for the future of technologies and engineering [1]. Nanocomposites come in a variety of structural forms, the most common of which are dispersions of nanoparticles or oriented distributions of nanofibers inside matrices of different types. Materials comprising composite nanoparticles often present a more sophisticated form of nanocomposites, from both synthetic and functional standpoints. A typical form of such materials would be a powder consisting of individual nanoparticles, each of which is multiphasic in composition. Taking advantage of the original definition of nanomaterials, according to which any structural unit in a physical system with at least one spatial dimension under 100 nm classifies the given system as a nanostructure [2], another form of such nanocomposites is that composed of microscale particles either embedded, or decorated on the surface, with nanoparticles. One such type of nanocomposite was synthesized and analyzed for its selected physicochemical and biological properties in the course of this study.

Nanoparticles that this nanocomposite comprised were composed of hydroxyapatite (HAp), the synthetic version of the inorganic crystalline component of mammalian bones and teeth. Although traditionally used as a filler for bone defects because of its chemical and crystallographic similarity to biogenic apatite, the application repertoire of HAp has been continually expanding over the past years and decades [3]. In the late 1970s, the first studies on loading HAp with organic molecules and measuring their release were reported [4,5] but, as ever [6], it would take a couple of decades before the interest in the use of HAp as a drug delivery vehicle would become mainstream [7]. This period that served as a precursor for the entrance of HAp to the drug delivery arena was paralleled by the long history of harnessing the high efficiency with which HAp binds proteins and nucleic acids. This binding affinity was first employed to separate DNA in chromatographic columns in the late 1960s [8] and then to deliver it intracellularly in the early 1970s [9], at which point HAp started to be used widely as a non-viral transfection agent. The overall adsorption and ion-exchange capacity of HAp is, in fact, on a par with that of zeolites, bentonites, and other highly adsorptive clays [10], enabling the achievement of comparatively high drug loading efficiencies. Furthermore, owing to its sparse solubility under neutral conditions, and its prompt dissolution at pH < 3, HAp nanoparticles allow for the endosomal escape of their molecular cargo, thereby facilitating its effective delivery to the target organelle following intracellular localization [11].

Although HAp is characterized by superior cell uptake efficiencies and excellent biocompatibility and bioactivity profiles, one significant drawback of HAp as a drug delivery vehicle comes from its inability to entrap organic molecules, regardless of how small they get, inside its crystalline lattice [12]. Thanks to its “zwitterionic” alternation of highly charged divalent calcium ions and trivalent phosphates on the surface, HAp does have a propensity to bind organic molecules via physisorption [13], but such molecules tend to undergo a burst release soon after the contact of the particles with the solvent [14], thus disabling the sustained release, which is a *sine qua non* for most drug delivery carriers. To stabilize the drug load in HAp, two strategies are commonly employed. One is based on controlling the aggregation of fine particulates and capturing small molecules inside the micropores forming inside such aggregates [15,16,17]. Another frequently implemented strategy is that of coating drug-loaded HAp with polymers, ideally in the form of narrowly dispersed and individualized core/shell particles [18,19,20]. While the first strategy usually leads to the formation of oversized aggregates that are unstable in the colloidal form, the second strategy can preserve the colloidal stability while providing an additional drug reservoir, as well as a surface that can be covalently functionalized, unlike that of an ionic crystal, which HAp is. This strategy is, furthermore, natural to a polymer chemist because polymers have been traditionally used to protect and release drugs at low rates by no means other than by their encapsulation or entrapment. There are, of course, scenarios where HAp nanoparticles are added to polymeric scaffolds, but even then, as it is the case with the core/shell colloidal spheres composed of polymer-coated HAp, HAp nanoparticles are mostly immersed inside the matrix and only minimally exposed on the surface [21,22,23].

However, HAp nanoparticles have a superior bioactivity compared to that of virtually all polymers, and shielding them from the contact with the physiological environment takes a toll on how positive the response of the body to these composite particles can be. As the polymeric shell gradually degrades upon the prolonged exposure to physiological fluids, cells and enzymes, HAp nanoparticles do eventually become exposed as well, but their bioactivity is of the most vital importance in the earliest stages of tissue/material interaction, when the immune response is triggered and the difference between the acceptance and the rejection of the material gets established by the organism. For that reason, it can be hypothesized that the reverse type of HAp/polymer composite particles, existing in the form of HAp-coated polymer instead of the other way around, may have a greater medical potential. In one such composite nanoparticle, nanostructured HAp on the surface would endow the particle with an excellent osteoconductivity in bone tissues and an excellent bioactivity in general tissues. At the same time, microporosity consequential to the HAp coating being composed of a conglomeration of nanoparticles may allow for the unimpeded influx of water toward the polymeric core and the release of the therapeutic cargo into the environment. Assessing whether these hypotheses regarding the drug release kinetics and bioactivity, as measured by the osteoblastic cell affinity, are true was the central objective of this study. Despite the handful of studies reporting on the synthesis and characterization of HAp-coated polymeric spheres, starting with the work by Tamai and Yasuda from 1998 [24] and then, after a decade-long incubation period, continuing throughout the late 2000s [25,26,27] and 2010s [28,29], these questions pertaining to the drug release kinetics and bioactivity have remained open.

## 2. Materials and Methods

### 2.1. Synthesis

HAp nanoparticles were synthesized from ionic precursors by chemical precipitation from an alkaline solution. Specifically, to precipitate HAp, 150 mL of 0.06 M aqueous solution of ammonium dihydrogen phosphate, NH_4_H_2_PO_4_, containing 7 mL of 28% ammonia, NH_4_OH, was added dropwise to the same volume of 0.1 M calcium nitrate, Ca(NO_3_)_2_, supplemented with 15 mL of 28% NH_4_OH. During the mixing of the reagents, the calcium solution was kept on a plate heated to 50 °C and stirred vigorously with a magnetic stir bar at the rate of 400 rpm. After the mixing of reagents was complete, the colloidal sol was heated up to the boiling point (circa 100 °C) and then removed from the heating pad and allowed to cool in the ambient air. Stirring was suspended and the suspension was aged at room temperature overnight. After the given time, the suspension was centrifuged for 3 min at the rate of 3500 rpm, after which the supernatant was decanted. The precipitate was washed with distilled water and the sequential centrifugation, decantation, and washing procedure was repeated.

To prepare poly(lactic-*co*-glycolic acid) (PLGA) spheres coated with HAp nanoparticles (PLGA@HAp), dried HAp nanoparticles were redispersed in water and the pH of the resulting suspension was adjusted to 7. PLGA (M_w_ = 45–75 kg/mol; co-monomer ratio 50:50, Durect) was dissolved in dichloromethane by handshaking the solution for 1 min. Subsequently, the polymer solution was added drop by drop to the HAp suspension at different volume ratios, e.g., 1:10 and 1:5. The entire mixture was hand-shaken for 1 min at the room temperature and was then left undisturbed for 1 min for the precipitate to settle. The precipitate was washed and dried at 25 °C. To synthesize bare PLGA particles, the same procedure was repeated, but without any HAp nanoparticles.

**Figure 1:**
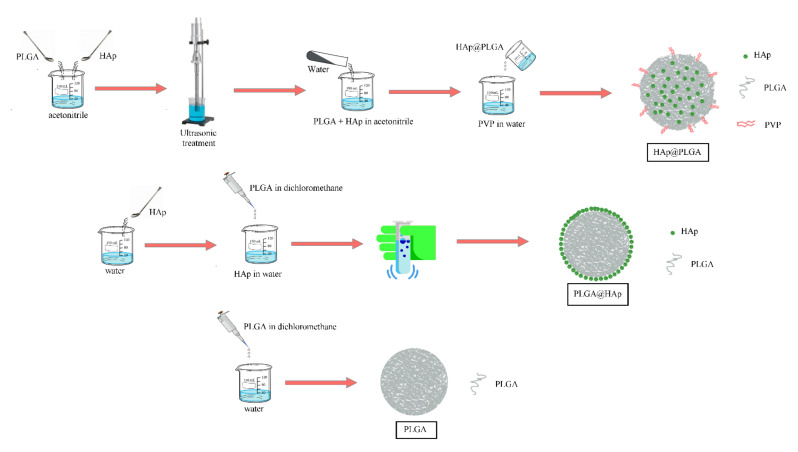
Schematic illustration of the steps in the syntheses of HAp@PLGA, PLGA@HAp, and PLGA particles.

The method for the preparation of PLGA-coated HAp (HAp@PLGA) was adapted from an earlier work [30]. PLGA in the amount of 900 mg was added to 40 mL of acetonitrile, after which HAp powder was added to the mixture and ultrasonically dispersed for 2 min. Immediately after that, 80 mL of water was added abruptly to initiate the precipitation of the polymer and form HAp@PLGA particles. The resulting suspension was added dropwise to 200 mL of 2 wt.% polyvinyl pyrrolidone (PVP) solution in water to stabilize the surface of the particles. Sedimentation was induced by a 2 h long centrifugation at 4000 rpm and 8 °C to prevent irreversible coalescence of the particles. All precipitates were dried in the air. A schematic illustration of the protocols used for the synthesis of PLGA, PLGA@HAp, and HAp@PLGA particles is shown in Figure 1.

### 2.2. Characterization

Scanning electron microscopy (SEM) studies were carried out on a JSM 6320F-FESEM (JEOL, Peabody, MA, USA) operated at a 4.2 kV voltage with an 8 μA beam current. Prior to imaging, powder samples deposited on clean aluminum stubs using carbon tape were sputter-coated with gold to reduce charging artifacts. X-ray diffraction (XRD) was performed on a D2 Phaser diffractometer (Bruker, Billerica, MA, USA). Polychromatic Cu was used as the irradiation source, and K_β_ line was stripped off with an inbuilt filter, while K_α2_ line was stripped off manually. The step size was 0.01°, with 1 s of sample irradiation per step. The zeta potential of particle suspensions in 150 mM KCl was measured using a Zetasizer Nano-ZS dynamic light scattering (DLS) device (Malvern, Worcestershire, UK). The Smoluchowski model was applied to convert the electrophoretic mobility values to ξ-potentials:

(1) $$\xi =\frac{\eta \nu }{\varepsilon E}=\frac{\eta \mu }{\varepsilon }\;\left[\mathrm{Volt}\right]$$

where ν is the electrophoretic velocity [cm/s], η the viscosity coefficient of the medium [dyn-sec/cm^2^], μ the electrophoretic mobility [μm·cm/V·s], ε the dielectric constant of the medium (circa 80 for water), and E the gradient of the electric field applied [V/cm].

### 2.3. Drug Release

The present study stems from a project focused on the use of HAp for the controlled delivery of antibiotics in the treatment of osteomyelitis. Naturally, therefore, the drug chosen to assess the release kinetics was an antibiotic, specifically vancomycin, a 1449 Da glycopeptide molecule effective against methicillin-resistant *Staphylococcus aureus*, the major causative agent of osteomyelitis [31]. HAp nanoparticles were loaded with vancomycin (MP Biomedicals) by adding the antibiotic at 2.4 wt.% to freshly prepared HAp powder in a 50 mL Falcon tube, mixing the suspension on a digital vortex mixer (Fisher Scientific, Waltham, MA, USA) for 5 min at 2000 rpm. This drug-loaded HAp was then used in the procedure for the formation of PLGA@HAp particles.

Drug release was measured by taking 1 mL of aliquots from the 50 mL phosphate buffered saline (PBS) medium in which the vancomycin-loaded nanocomposite powder was placed. PBS was kept inside closed conical tubes and incubated at 37 °C and 50 rpm. The volume of the aliquoted solution was replaced with fresh PBS, and consequential changes to the drug concentration in the supernatant were taken into account. The vancomycin concentration was determined by measuring the absorbance on a UV/Vis spectrophotometer (Nanodrop 2000, Thermo Scientific, Waltham, MA, USA) at λ = 200 nm. Release assays were performed in triplicate.

To delineate the release mechanism, the drug release profiles were fitted to the Korsmeyer–Peppas, Hixson–Crowell, and Higuchi models [32]. The Korsmeyer–Peppas model assumes the validity of the following equation, where M_t_ is the amount of the drug released by the time t, M_0_ is the total amount of the drug entrapped in the carrier, k_K-P_ is the release rate constant derivable from the *y*-axis intercept, and n is the Korsmeyer–Peppas exponent calculated from the slope and being indicative of the mechanism of the release:

log(M_t_/M_0_) = logk_K-P_ + nlogt (2)

The Hixson–Crowell model assumes the validity of the following equation, where M_t_ is the amount of the drug released by the time t, M_0_ is the total amount of the drug entrapped in the carrier, and k_H-C_ is the release rate constant derivable from the slope of the curve:

M_0_^1/3^ − M_t_^1/3^ = k_H-C_t (3)

Finally, the Higuchi model assumes the validity of the following equation, where M_t_ is the amount of the drug released by the time t, in percentages, and k_H_ is the release rate constant derivable from the slope of the curve:

M_t_ = k_H_t^1/2^ (4)

### 2.4. Cell Culture

Cell culture experimentation was performed on the MC3T3-E1 subclone 4 cell line purchased from the American Tissue Culture Collection (ATCC, Rockville, MD, USA). MC3T3-E1 are murine calvarial preosteoblastic cells, and they were cultured in Alpha Minimum Essential Medium (α-MEM; Gibco) supplemented with 10% fetal bovine serum (FBS, Invitrogen) and an antibiotic/antimycotic additive (Gibco). The cultures were incubated at 37 °C in a humidified atmosphere containing 5% CO_2_. The medium was replaced every 48 h. The cultures were regularly examined under an optical microscope to monitor growth and possible contamination. Weekly, the cells were passaged by detaching them from the surface of the cell culture flask (Greiner Bio-One) using 0.25 wt.% trypsin. Each passaging involved washing, centrifugation (1000 rpm × 3 min), resuspension in 10 mL fresh media, and subculturing of the cells at a 1:7 volume ratio.

To visually assess the interaction between the MC3T3-E1 cells and the different particles, the cells were seeded on glass cover slips fitted inside 24-well plates at a density of 6 × 10^4^ cells per well and in 400 μL of the cell culture medium. Near confluence, cells were treated with 2 mg/cm^2^ of HAp@PLGA or PLGA@HAp particles and 50 μg/mL ascorbic acid as the osteoblastic differentiation agent, and incubated for 7–10 days. Alpha-MEM supplemented with 50 μg/mL ascorbic acid was replenished every 48 h.

The accumulation of the collagen matrix in differentiated MC3T3-E1 cells is maximal after 7 days of culture [33]. After 7 days of incubation with the particles, cells grown on the cover slips in 24-well plates were stained for collagen type I, nucleus, and HAp. The staining protocol began with washing the cells with PBS and fixing them for 15 min in 3.7% paraformaldehyde. In the next step, the cells were washed first with PBS triply, for 5 min each, and then with the blocking solution (1% bovine serum albumin, 0.1% Triton X-100 in PBS) doubly, for 5 min each. The cells were then blocked and permeabilized in the blocking solution for 1 h. To stain for collagen, the cells were incubated with 10 μg/mL rabbit anti-collagen-type-1 (Abcam, Cambridge, UK) as the primary antibody in the blocking solution for 1 h. The cells were then washed with PBS triply, for 10 min each, and incubated with 10 μg/mL AlexaFluor 555 goat anti-rabbit IgG (Invitrogen, Waltham, MA, USA) as the secondary antibody, 20 μg/mL 4′,6-diamidino-2-phenylindole dihydrochloride (DAPI, Invitrogen, Waltham, MA, USA) as the nuclear counterstain, and 2 μM calcein AM as a HAp-staining agent, all in the blocking solution for 1 h. The cells were then washed with PBS triply, for 5 min each. Cover slips containing the fixed and stained cells were mounted onto microscopy slides using VECTASHIELD HardSet mounting medium and nail polish. Cells were imaged on a Nikon T1-S/L100 confocal optical microscope at 60 x magnification in oil. All the experiments were performed in triplicate.

Differentiation of MC3T3-E1 preosteoblasts into osteoblast-like cells leads to the formation of a mineralized extracellular collagen matrix. It also entails the expression of genes associated with the osteoblastic phenotype. After the incubation period of 10 days, cell lysis, reverse transcription (Bio-Rad), and qPCR (Applied Biosystems, StepONEPlus) were performed using the Fast SYBR Green Cells-to-CT kit (Ambion, Austin, TX, USA), as per the manufacturer’s instructions. Each experiment was performed in quadruplicate, while each experimental replica was analyzed for mRNA expression in triplicate (*n* = 4 × 3). The expressions of one housekeeping gene, β-actin (*ACTB*), and three osteogenic markers, mouse type I procollagen (*Col I*), osteocalcin (*BGLAP*), and *Runx2,* were analyzed. The primer pair sequences used in the analysis are listed in Table 1. Raw real-time PCR data were analyzed using the ΔΔCt method. All data points pertaining to individual genes were normalized to the expression levels of *ACTB* as the housekeeping gene.

## 3. Results and Discussion

PLGA, the polymer used here in combination with HAp, is commonly employed as a drug delivery carrier [34,35,36], or the main component of tissue engineering constructs [37,38,39]. In the body, it degrades to lactic and glycolic acid monomers, which are incorporated in natural metabolic pathways or excreted without complications [40]. The greatest risk entailing the application of PLGA as a biomaterial, in fact, comes from the local increase in acidity consequential to the acidic nature of both lactic and glycolic acids (pKa ~3.8 for both), which may trigger an oft-observed [41] acute inflammatory response, or favor the proliferation of not only acidophilic bacterial species, such as lactic acid fermenters, but also neutrophils, such as *Staphylococcus aureus* [42] or *Pseudomonas aeruginosa* [43], under opportunistic conditions possibly related to local microbiome disruption in some scenarios. In that sense, the combination of PLGA with HAp comes natural because of the ability of hydroxyls released by the dissolution of HAp to compensate for the acidic products from the degradation of PLGA.

**Figure 2:**
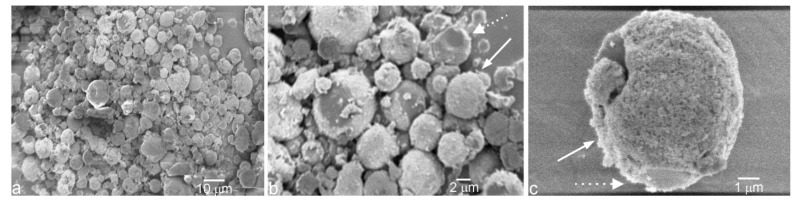
Scanning electron micrographs of PLGA@HAp particles at different magnifications: 1000× (**a**), 3000× (**b**), and 9500× (**c**). The dashed-line arrows in (**b**,**c**) point at bare patches on the PLGA particle surface, while the full-line arrows point at fully HAp-coated regions on the PLGA particle surface.

By attracting HAp nanoparticles to the oil–water interface and then allowing the polymeric phase to grow out of it, the method for the synthesis of HAp-coated PLGA spheres (PLGA@HAp) adopted in this study is partially based on the stabilization of Pickering emulsions [44]. Optimizations of the synthesis method were required to obtain uniformly sized microspheres. Figure 2 shows such relatively narrowly dispersed microsized PLGA spheres coated with finer HAp nanoparticles. The diameter of the composite particles was in the 3–8 μm range. The absence of HAp nanoparticles not associated with the polymeric spheres indicated a good degree of attraction between the two phases comprising the composite particles. The volume ratio between the polymer solution and the HAp dispersion proved decisive for defining the particle properties. Specifically, as this ratio increased, so did the average microsphere size. The oblation of the particle surface and its smoothness also increased in direct proportion with this ratio. To gain insight into the biological potency of the PLGA@HAp system, it was of pivotal importance to compare its properties against the opposite and more common system consisting of HAp nanoparticles embedded inside PLGA spheres. These composite particles were characterized in detail in our earlier work [45], and the most critical difference between the two composite systems was the lower size of HAp@PLGA particles, averaging at ~250 nm, compared to those of PLGA@HAp.

**Figure 3:**
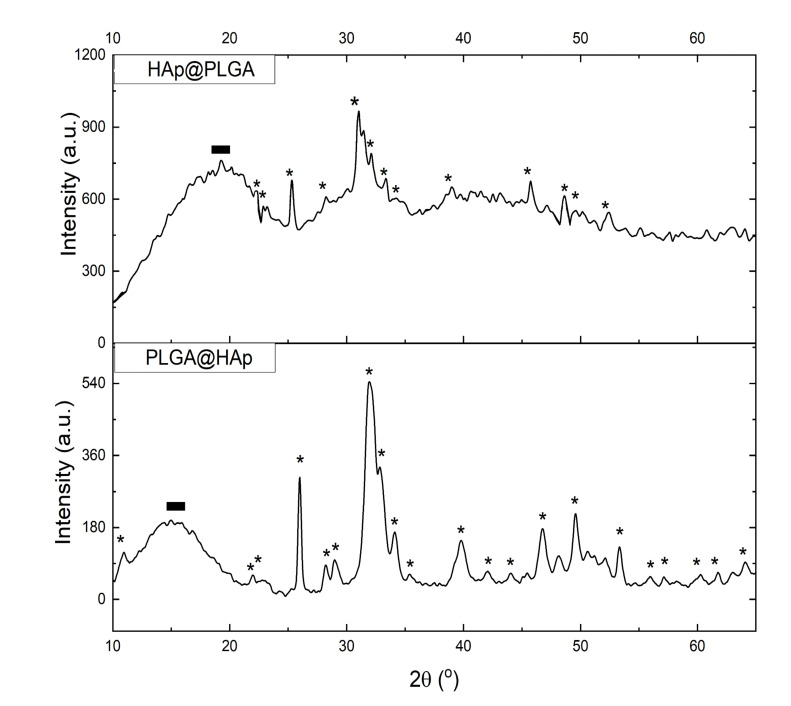
X-ray diffractograms of HAp@PLGA and PLGA@HAp particles. The most intense reflections originating from HAp are topped with asterisks, while the low-angle diffuse reflection originating from amorphous PLGA is denoted with black rectangles.

Regardless of whether HAp nanoparticles were trapped inside PLGA spheres or decorated their surface, they were detectable using XRD. Thus, as seen in Figure 3, all the major diffractometric reflections typifying HAp were present in the XRD patterns of both PLGA@HAp and HAp@PLGA powders. Naturally, the composite exposing HAp nanoparticles on the surface displayed a less-noisy signal and more distinct peaks originating from this inorganic phase. No significant difference in the integral breadths of HAp reflections existed depending on whether they originated from HAp@PLGA or PLGA@HAp, as expected from their identical crystallinity in these two systems. Had HAp nanoparticles been crystallized in different environments containing PLGA, a difference in crystallinity would be expected. However, since the PLGA spheres were precipitated in the presence of prefabricated HAp nanoparticles, rather than *vice versa*, only the difference in crystallinity and other microstructural features of PLGA could be expected. This difference came in the form of a shift of the diffuse reflection of PLGA to higher diffraction angles when PLGA was exposed on the surface of the composite nanoparticles. Thus, while this diffuse reflection peaked at 2θ = 15.0° in the XRD pattern of PLGA@HAp, it peaked at 19.2° for HAp@PLGA. As per the Bragg relation, *nλ = 2dsinθ*, where *n* is the order of reflection, *λ* is the wavelength of the incident radiation, and *d* is the interplanar distance, higher diffraction angles correspond to lower interplanar distances, meaning that the method for the synthesis of PLGA forming HAp@PLGA composite particles leads to more tightly packed polymeric chains at the level of the unit cell in comparison to those comprising PLGA@HAp.

**Figure 4:**
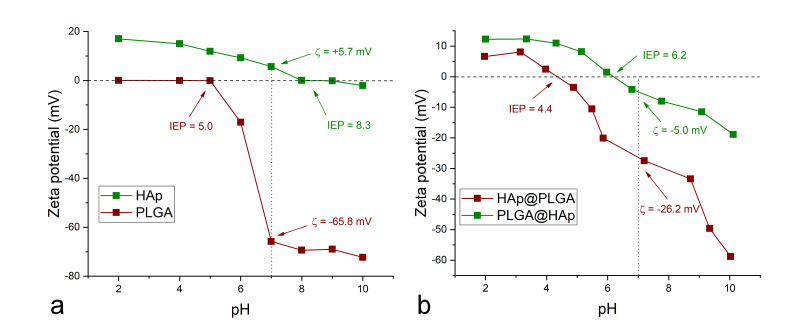
Comparison of zeta potential vs. pH titration curves for PLGA and HAp particles (**a**) and for PLGA@HAp and HAp@PLGA particles (**b**). Zeta potential values obtained under the neutral pH conditions and isoelectric points (IEPs) for PLGA and HAp (**a**) and for PLGA@HAp and HAp@PLGA (**b**) are denoted in the graphs.

The forces binding HAp on the surface of PLGA are weak, primarily van der Waals forces, but the binding process is most likely driven by the electrostatic attraction. This is evidenced from the zeta potential measurements, which indicated a significant difference in the surface charge magnitude and sign between HAp and PLGA, under neutral conditions (pH 7 at 25 °C). Titration curves over the pH range of 2–10, shown in Figure 4a, were, in fact, constructed prior to the synthesis in order to delineate the pH conditions under which the electrostatic attraction between PLGA and HAp would be maximal. Such conditions were found at pH 7, where the zeta potential of HAp nanoparticles was slightly positive (+5.7 mV), while the zeta potential of PLGA spheres was highly negative (−65.8 mV). Although the significantly higher isoelectric point (IEP) of HAp compared to that of PLGA−8.3 vs. 5.0, respectively, indicated the relatively wide window of pH values within which the charges on the two phases are opposite, ranging from 5.0 to 8.3, the best electrostatic conditions for interaction between the two phases existed under the neutral conditions, where the greatest difference in the opposite charge magnitude was measured. Such conditions were, for this reason, employed in the synthesis of PLGA@HAp particles. It is probable that the same electrophoretic principle could be utilized to derive conditions for the coating of HAp around polymeric particles with different chemical compositions.

In addition to proving useful in preparation for the synthesis of composite particles, zeta potential measurements are useful after the synthesis as well, specifically to indirectly demonstrate the coating of one phase by the other [46]. Zeta potential curves displayed in Figure 4b provide one such indirect confirmation of the successful coating of HAp by PLGA in HAp@PLGA and of PLGA by HAp in PLGA@HAp. This can be seen from the high level of semblance between the zeta potential titration curves of HAp and PLGA@HAp, as well as of PLGA and HAp@PLGA. Naturally, the phase exposed on the surface of the composite particle will determine its zeta potential characteristics, as opposed to the phase shielded inside it. The full coverage of HAp nanoparticles in HAp@PLGA is indirectly evidenced by the shift in the IEP to lower values, away from the IEP of HAp, specifically from 5.0 to 4.3, with the transition of PLGA to HAp@PLGA (Figure 4). In contrast, the incomplete coverage of PLGA spheres by HAp nanoparticles is indirectly evidenced by the shift in the IEP of PLGA@HAp nearer to the IEP of pure PLGA, specifically from 8.3 to 6.2 (Figure 4).

**Figure 5:**
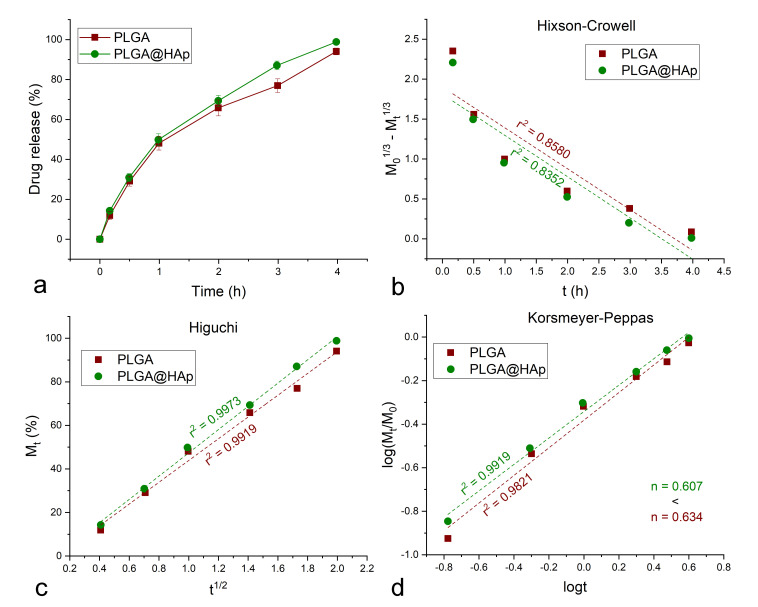
Vancomycin release profiles of PLGA spheres and PLGA spheres coated with HAp nanoparticles (PLGA@HAp) (**a**) and their fits with the Hixson–Crowell (**b**), Higuchi (**c**), and Korsmeyer–Peppas (**d**) kinetic models. Error bars in (**a**) represent average standard deviations deduced for similar samples under identical release conditions.

The analyses of the drug release kinetics showed that more than 95% of the drug load was released within about 4 h, with the release being just slightly faster from PLGA@HAp than from PLGA. More specifically, 94% of the overall drug load was released after 4 h from PLGA and 98.8% from PLGA@HAp. The greatest difference in the released amount was noted by the end of the third hour, when 76.9% of the drug was released from PLGA and 87.1% from PLGA@HAp. Vancomycin was chosen for the model drug in these studies for two reasons. First, it is a glycopeptide antibiotic for which we noticed a very interesting synergy in our earlier antibacterial studies. Namely, all by itself, it showed no effect against biofilm-forming Gram-negative bacteria, such as *P. aeruginosa*, but when delivered in conjunction with the precursor of HAp in the form of amorphous calcium phosphate, its activity became significant [47]. Therefore, we aim to continue to explore its synergy with HAp and HAp-based composite materials. Secondly, vancomycin is a hydrophilic antibiotic and is convenient to work with in aqueous media, along with being adsorbed onto HAp more effectively than its hydrophobic analogues while remaining loadable into PLGA. A number of studies have reported on the successful loading and delivery of vancomycin with the use of PLGA spheres [48,49,50]. Overall, the drug release results confirm that the addition of the HAp shell around the PLGA spheres does not impede the release from the polymer. Quite contrarily, by taking a portion of the drug upon itself, the HAp shell even speeds up the release of the drug compared to bare PLGA spheres, in agreement with the aforementioned propensity of HAp for the burst release of the drug, as opposed to a more delayed release expected from the polymeric phase. Nonetheless, the lack of a distinct burst release period in the first hour of the release, and the approximate overlap of the release curves corresponding to PLGA and PLGA@HAp (Figure 5a) indicates that most of the drug enters the polymeric phase and is released from it, while the portion of the drug that HAp has taken upon itself is comparatively small.

In a previous study where PLGA spheres were coated with HAp using Kokubo’s method of immersion in simulated body fluid [51], a more drastic increase in the release rate was observed after the addition of the HAp surface layer, which was hypothesized to have been due to the comparatively weaker bonding of the carboxylic moieties of the drug molecules to the calcium ions of HAp than to the polymeric chains [52]. Similar formation of weaker and more dissociable bonds with the drug following the addition of the HAp phase should be a definite factor favoring the higher rate of release from PLGA@HAp particles than from pure PLGA. However, the more tortuous path of release of the drug molecules from the interior of the polymeric spheres, which must physically erode to allow the escape of the drug, than from HAp residing on the particle surface and holding the drug load by mere physisorption, is an equally important factor predisposing PLGA@HAp particles to the faster release of vancomycin as compared to PLGA particles. Moreover, the mildly accelerated rate of drug release from PLGA@HAp particles as compared to that from PLGA eliminates the possibility that alkaline HAp neutralized the acidic byproducts of the autocatalytic degradation of the polymer, in which case, the opposite effect, namely that of reduced degradation and drug release rates after the addition of the HAp layer, would have been observed.

Comparison of the fits of the kinetic release data with different theoretical models showed different levels of agreement. In particular, the fit with the Hixson–Crowell model (Figure 5b) was relatively weak (r^2^ = 0.84 − 0.86) and knowing that this model applies to formulations where the carrier degradation is the driving force behind the drug release, it could be deduced that the release is mainly governed by the diffusion of the drug from the dosage form, as expected from the relatively slow degradation of PLGA and HAp in comparison with the completion of the release in ~4 h (Figure 5a). The fact that the release of the drug proceeds primarily by diffusion from the carrier is confirmed by the excellent fits with both the Higuchi model (r^2^ > 0.99) (Figure 5c) and the Korsmeyer–Peppas model (r^2^ > 0.98) (Figure 5d), both of which apply to diffusion-controlled release mechanisms.

With the addition of HAp onto PLGA spheres, the value of the Korsmeyer–Peppas coefficient, *n*, decreased from 0.634 to 0.607 (Figure 5d), indicating a more Fickian diffusion through which vancomycin gets released from the composite particles and into the solution. The Korsmeyer–Peppas coefficient, in general, is indicative of the release mechanism, suggesting an ideal, Fickian diffusional process for *n* = 0.45 in spherical particles [53]. With an increase in *n*, the diffusion becomes less Fickian and more anomalous. The fact that the release is more Fickian in PLGA@HAp than in PLGA is expected because in PLGA@HAp, the drug is distributed, albeit unequally, between two phases, namely PLGA and HAp. Although a large percentage of the drug can readily diffuse out of the polymeric phase through the pores in the particle structure, the polymeric network does impose some hindrance to the release of a portion of the drug molecules, resulting in the deviation from the ideal Fickian scenario. This portion is naturally lower in PLGA@HAp than in PLGA because a small amount of the drug does not get captured by the polymer during its solidification, but rather remains confined to the HAp shell. Since this drug binds to HAp via weak physical bonds, its release from HAp is more driven by the concentration gradients between the HAp particle surface and the solution than it is the case for the drug confined to the polymeric core. The trend toward a more Fickian diffusion with the addition of HAp indirectly reflects the different mechanism by which the drug is loaded onto the two phases, involving adsorption in the case of HAp and physical entrapment in the case of PLGA.

**Figure 6:**
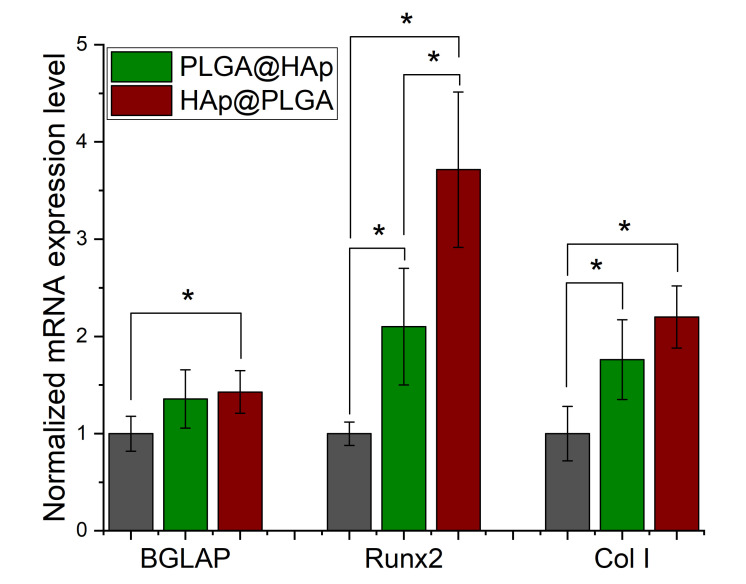
The comparative effect of PLGA@HAP and HAP@PLGA particles on the mRNA expression of three different osteogenic markers—*BGLAP*, *Runx2*, and *Col I*—in osteoblastic MC3T3-E1 cells. mRNA expression was detected using quantitative RT-PCR relative to the expression of *ACTB* as the housekeeping gene. Data normalized to the expression of *ACTB* are shown as averages, with error bars representing the standard deviation. Genes significantly (*p* < 0.05) upregulated, with respect to the control group, are marked with *. No genes were downregulated with respect to the control group.

MC3T3-E1 cells are fibroblastic in origin, but can be differentiated into an osteoblastic lineage when exposed to ascorbate and phosphate anions [54]. This differentiation proceeds with the activation of specific genes and metabolic pathways, which can be measured in response to the therapy and compared with negative and/or positive controls [55]. Three distinct osteogenic markers were analyzed in this study, including the mineralization inductor osteocalcin (*BGLAP*), the extracellular matrix protein collagen type I, and the transcription factor *Runx2*, all at the level of expression of their genes, not proteins. Based on the results of the gene expression analysis, the cells, first of all, reacted to the exposure to particles *per se*, regardless of their composition. This is seen from the expression of all three osteogenic markers being significantly higher in 5 out of 6 cases for cells challenged with either HAp@PLGA or PLGA@HAp particles as compared to the cells grown on cell culture plastics and not treated with any powders (Figure 6). However, contrary to the expectation, between the two cell populations treated with the composite particles, a higher osteogenic activity was detected in cells challenged with HAp@PLGA than in cells treated with PLGA@HAp.

**Figure 7:**
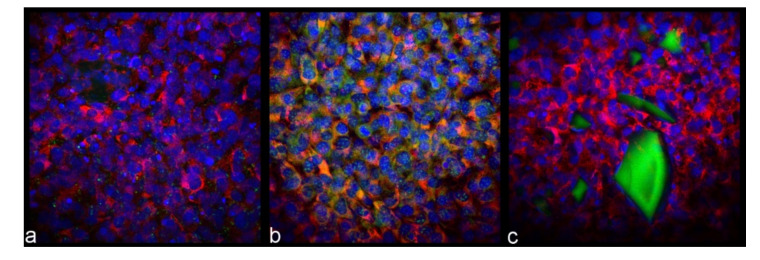
Immunofluorescent optical micrographs of control osteoblastic MC3T3-E1 cells (**a**) and osteoblastic MC3T3-E1 cells challenged with 2 mg/cm^2^ HAp@PLGA (**b**) or PLGA@HAp (**c**) particles for 7 days.

The higher gene expression of osteoblastic markers in cells treated with the composite particles, wherein HAp nanoparticles were buried inside them rather than exposed on the surface, was corroborated by the immunofluorescent analysis of the cell morphologies (Figure 7). According to the results of this analysis, a greater density and finer distribution of mineral particles was observed in the osteoblastic cells challenged with HAp@PLGA than in those challenged with PLGA@HAp. It should be noted here that one portion of the green signal in the immunofluorescent optical images shown in Figure 7 originates from the endogenous mineral nodules formed by the cells after their differentiation into the osteoblastic phenotype, while the other part is exogenous in nature, coming from the HAp nanoparticles added externally to the culture. Because the majority of the green signal in cultures treated with PLGA@HAp is confined to PLGA@HAp particle agglomerates (Figure 7c), it can be concluded that most of the mineral presence in these cultures is exogenous in origin. In contrast, the signal appears to be more endogenous in cultures treated with HAp@PLGA (Figure 7b), given that HAp nanoparticles here are, for the most part, shielded from the contact with the staining agent. That the majority of the green signal is supposed to come from the endogenous mineral is, in fact, expected from the results of the gene expression analysis.

The greater degree of dispersion of HAp@PLGA particles compared to that of the PLGA@HAp particles can be explained by the surface charge effects. Specifically, PLGA@HAp particles demonstrated a more pronounced tendency for aggregation as the result of their zeta potential values being within the ±15 mV instability window under the physiological conditions, as opposed to HAp@PLGA particles, which exceeded the ±30 mV threshold of permanent stability under the same conditions (Figure 4b). This effect is evidenced from the optical micrographs in Figure 7, which show a greater degree of dispersion of HAp@PLGA particles inside and around the cells, as opposed to a greater aggregation degree exhibited by PLGA@HAp particles. Still, this aggregation effect refers solely to the exogenous HAp and HAp-containing particles and does not take into account HAp particles formed endogenously, by the osteoblastic cells, which undoubtedly contribute to the green signal, especially in cells challenged with HAp@PLGA, but also in control cells and cells treated with PLGA@HAp to a lesser extent.

The findings of the gene expression and immunofluorescent imaging analyses tie back to our earlier work [56], in which it was demonstrated that exposing osteoblastic cells to the bone mineral hampers their bone-production activity, while depriving them from the contact with the mineral stimulates this activity. Apparently, homeostatic mechanisms governing the cell metabolism ensure than the sensation of a product of this metabolism in the cell interior, or the surrounding area, is met with the reduction in its activity, contrasting with a less realistic scenario, in which the cell would sense the presence of a product of its metabolism in or around itself and create even more of it. The absence of such products in or around the cell is expected to be met with a boost in the metabolic activity of the cell, which in this case corresponds to a greater degree of mineral production. This is a simple consequence of the autopoietic nature of biological systems [57,58,59], typified by the perpetuation of negative, self-regulating feedback loops that lead to complex optimizations and dynamic equilibria and by the suppression of their positive analogues, which permanently threaten to send the system down destructive autocatalytic paths. To that end, the findings of this study oppose its initial hypothesis, which was that depositing a HAp shell around a polymeric particle would stimulate the osteogenic activity of the bone cells relative to the opposite scenario involving composite particles whose polymeric shells are coated around the HAp cores instead.

**Figure 8:**
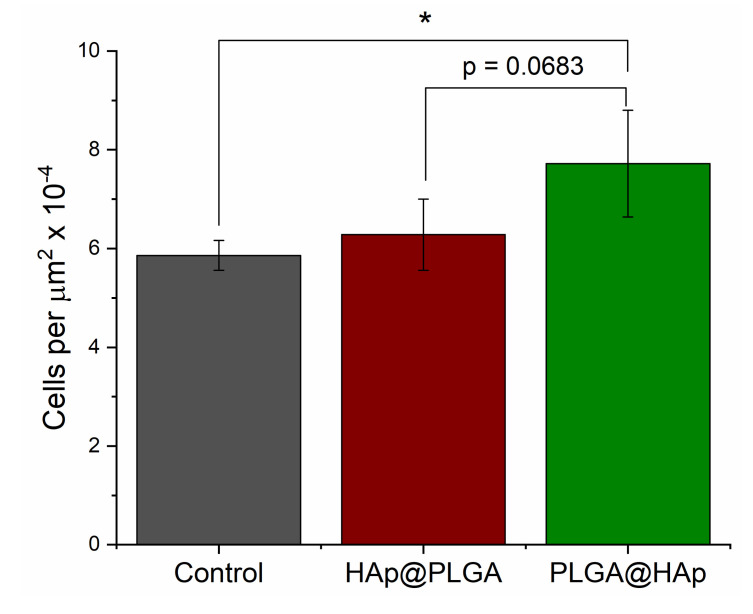
MC3T3-E1 cell density per surface area unit for the control population and populations challenged with 2 mg/cm^2^ HAp@PLGA or PLGA@HAp particles, as determined by the proliferation assay after 7 days of incubation. Genes significantly (*p* < 0.05) upregulated, with respect to the control group, are marked with *.

The osteogenic, bone-building activity of the cells and their proliferation are typically antagonistically related to one another [60]. In other words, when a cell undergoes differentiation to a particular phenotype, or activates the metabolic pathways resulting in the production of mineral particles and the collagenous boney tissue matrix, its mitotic and proliferative activities are diminished. This implies that even though the genetic activity of osteoblastic cells treated with PLGA@HAp becomes reduced compared to that of cells treated with HAp@PLGA, the adherence of the cells onto the biomaterial and their proliferation could follow a totally independent trend. To assess the features of this trend, a proliferation assay was carried out after 7 days of incubation of cells with different particles. The results demonstrated that the proliferation was indeed higher in the population brought into contact with PLGA@HAp particles, compared to both the control population and the population challenged with HAp@PLGA particles (Figure 8). This result is a direct effect of the greater bioactivity and wettability of HAp compared to those of PLGA, which leads to the promotion of a more intimate and proliferative interface between the PLGA@HAp material and the cells, if not of the augmented osteogenic activity and bone mineral production.

To sum up, the findings of the biological analyses counteract the central premise from which this study stemmed, which was that the more bioactive and osteoconductive surface layer of HAp on PLGA spheres would promote the osteogenic response at the cellular scale. However, with the HAp coating evidently increasing the proliferation rate of osteoblastic cells, the response could still be positive *in vivo*, as indicated by a number of studies where coating polymeric structures, including PLGA-based structures [61,62], with HAp promoted a better integration of the material with hard tissues in animals [63,64,65]. Similarly positive correlations were observed in some [66,67], but not all [68,69] soft tissues as well. However, as indicated by the results of this study, this positive osseointegration effect induced by HAp coatings may not be caused by the direct promotion of the osteogenic response through upregulation of genes in the osteoblasts. Rather, it is more likely consequential to other, more indirect effects, such as better tissue adhesion, mechanotransduction, vascularization, or simply the specifics of the multicellular response at the site of contact between the material and the tissue.

## 4. Conclusions

In search of the inspiration lost, a simple trick could be employed. It is that of changing the perspective by turning things upside down or inside out. What is prominent on the surface is thus being disregarded for a while and what is hidden from view is being brought to the forefront of our attention, with benefits of this approach being multifold. For example, to begin to perceive answered questions as unquestioned answers is to suddenly restore the glow of wonder and curiosity. So it may be with concepts commonly employed in the design of fine particles. One such concept has been that of encapsulating metallic and metal oxide nanoparticles inside polymeric microspheres, rather than *vice versa*, especially in the drug delivery field. In this study, we have investigated the pharmacokinetic and biological effects of the reversal of this paradigmatic concept achieved by coating PLGA microspheres with HAp nanoparticles, instead of the other way around. We confirmed that such systems, counterintuitively, could be formed by precipitating PLGA spheres as cores of these core/shell composite particles in the presence of the preformed HAp nanoparticles instead of the other way around. Zeta potential analyses assisted in defining the conditions under which the electrostatic attraction between PLGA spheres comprising the core and HAp nanoparticles comprising the shell would be maximal, but also for verifying the success of the coating process. The HAp shell did not impede the release of vancomycin from the particles; rather, its addition to PLGA spheres increased the drug release rate to a minor degree and made the mechanism of release less anomalous and more Fickian, as the result of the confinement of a portion of the drug to the HAp shell. Still, the virtual overlap of the profiles of release from decorated and undecorated PLGA spheres suggested that most of the drug content entered the polymeric core of PLGA@HAp particles and was released from it with a minimal influence of the HAp shell. Most interestingly, the decoration of PLGA spheres with HAp nanoparticles induced a lesser upregulation of the osteogenic markers, including osteocalcin, collagen type I, and *Runx2*, compared to that induced by the composite particles in which HAp nanoparticles were embedded inside the PLGA spheres. Naturally, it also led to a reduced amount of bone mineral detected in the cells differentiated into the osteoblastic phenotype compared to that observed in cultures treated with HAp nanoparticles coated with PLGA. This effect refuted the initial hypothesis of the study, which stemmed from the expectation that the presence of a more bioactive and osteoconductive surface layer of HAp on PLGA particles would enhance the osteogenic response of the cells. The more pronounced osteogenic response to particles that had HAp embedded inside them rather than exposed on the surface was explained by homeostatic mechanisms governing cell metabolism, which ensure than the sensation of a product of this metabolism in the cell interior or exterior, in this case bone mineral, is met with the reduction in the metabolic activity. The antagonistic relationship between proliferation and bone production was reiterated by demonstrating the higher proliferation rate of cells challenged with HAp-coated PLGA spheres than of those treated with PLGA-coated HAp. These results suggested that the overwhelmingly positive response of tissues and organisms to HAp-coated biomaterials for bone replacement is unlikely to be due to the direct promotion of new bone growth by the HAp coating. Rather, these positive effects are more likely consequential to a better tissue adhesion, mechanotransduction, osseointegration, and the overall more favorable multicellular response at the site of contact between the HAp-coated material and the tissue. These instructive fundamental insights notwithstanding, the practical benefits of utilizing HAp-coated PLGA spheres instead of PLGA-coated HAp in tissue engineering are less clear based on the results of this study and warrant further research into the subject. Nevertheless, the acquisition of informative basic findings has justified the approach based on the inside-out reversal of the particle content implemented in this study. Implicitly, the study reiterates the epistemic utility of turning material objects, alongside ideas and points of view, inside out and outside in. In even broader frames, it suggests that these and similar inversions of perspectives should endure, lest the wonder abate.

## Figures and Tables

**Table 1:** Primer pair sequences used in the real-time PCR analysis.

Gene	Forward 5′-3′ Primer	Reverse 5′-3′ Primer
*ACTB*	GGCCCAGAGCAAGAGAGGTATCC	ACGCACGATTTCCCTCTCAGC
*Col I*	GCGAAGGCAACAGTCGCT	CTTGGTGGTTTTGTATTCGATGAC
*BGLAP*	CTCACAGATGCCAAGCCCA	CCAAGGTAGCGCCGGAGTCT
*Runx2*	AAATGCCTCCGCTGTTATGAA	GCTCCGGCCCACAAATCT

## Data Availability

Data are available upon reasonable request.
